# Adopting information and communications technology in the control, prevention, and management of stroke: perspectives from patients and providers in Uganda

**DOI:** 10.3389/fstro.2024.1440047

**Published:** 2024-12-18

**Authors:** Prossy Kiddu Namyalo, Robert Setekera, Primrose Nakazibwe

**Affiliations:** ^1^Faculty of Social Sciences, Ndejje University, Kampala, Uganda; ^2^Faculty of Science and Computing, Ndejje University, Kampala, Uganda; ^3^Research and Innovation Department, Ndejje University, Kampala, Uganda

**Keywords:** ICT, eHealth, stroke, rural areas, patients, providers, Uganda

## Abstract

**Objective:**

The stroke burden in Uganda ranks ninth among the ten causes of death, a major cause of chronic illnesses, accounting for the top ten causes of hospitalization. This baseline study examined how mobile phones can improve the prevention, management, and treatment of stroke in rural Uganda.

**Methods:**

It was a cross-sectional study that utilized a mixture of methods. Quantitative data was collected from the districts' health information management system while qualitative data were from healthcare providers and patients/caregivers/survivors using a semi-structured guide. Quantitative data was analyzed descriptively while qualitative data was inductively analyzed through creating themes.

**Results:**

All participants supported the use of mobile phone interventions and suggested three major types of information to be included in this intervention: warning signs and indicators, underlying causes of stroke, and prevention measures. The challenges that might be faced in implementing this intervention are contextual, health system, and economic related.

**Conclusion:**

These baseline findings support the possibility of mobile phone intervention as an important instrument to improve stroke prevention, management, and treatment in rural Uganda. Challenges that might accompany the use of ICT have to be addressed as the intervention is designed.

## Introduction

Close to two decades ago, the WHO (World Health Assembly, 2005) urged member states to “develop the infrastructure for information and communications technology (ICT) for health has deemed appropriate to promote equitable, affordable, and universal access to their benefits, and to continue to work with information and telecommunication agencies and other partners to reduce costs and make eHealth successful” (Crotty et al., 2014). Indeed, the digitalization of healthcare is skyrocketing, and by 2026, digital health users are expected to exceed 200 billion (Nittas et al., 2023). Developed nations have several digital health tools including remote patient monitoring, education and coaching, and digital therapeutics assisting condition-specific factors. The tools have elements like medication reminders, activity trackers, recording blood pressure, and information (Patel, 2021). In chronic care, digital healthcare has been applied to a varied range of services including the provision of self-control and management (Villarreal et al., 2009; Koutkias et al., 2010; Gee et al., 2015), improving care access, and patient care outcomes (Beatty et al., 2013). Previous studies have indicated that patients with chronic ailments interfacing with health technologies can remotely interact with peers, have continuous access to relevant information, and can measure and track their health as well as engage in knowledge exchange (Nittas et al., 2023; Han and Lee, 2021). However, the majority of such studies were conducted outside Uganda and in urban settings.

Stroke remains one of the major public health concerns and the most devastating of all neurological and non-communicable diseases worldwide (Donkor, 2018). In Uganda, stroke ranks ninth among the 10 causes of death, a major cause of chronic illnesses, and accounts for the top ten causes of hospitalization (Namale et al., 2020; CDC, 2021). Stroke-related mortality varies considerably between stroke types, regions, and countries. For example, in Uganda, 30-day mortality was found to be 43% compared to 27 and 29.3% in Gambia and Tanzania respectively. Hospital-based studies in Uganda report stroke mortality at an estimated percentage of 30–40 in 1 month; a figure higher than 20% at the world level (Namale et al., 2020). Additionally, in Uganda, many stroke patients receive their initial care in Mulago National Referral Hospital situated in Kampala, a place with heavy traffic congestion, deemed far for upcountry patients coupled with poor transport means, causing more delays for stroke patients in need of early services (Kamwesiga et al., 2018a).

In Uganda, mobile phone applications are used to increase access to sexual, reproductive, and health information goods and services among university students (Nalwanga et al., 2021; Nuwamanya et al., 2020), maternal and child health (Musiimenta et al., 2020, 2021), HIV (Musiimenta et al., 2018), and Tuberculosis (Musiimenta et al., 2019) among others. Regarding Stroke, the interventions and related studies are focused on rehabilitation care with limited or no empirical evidence on stroke prevention and therapeutic (Kamwesiga et al., 2018a,b; Teriö et al., 2019). Moreover, most of the available evidence is from the users'/patients' perspectives. It can be urged that there is a lack of expert (health workers) involvement in those interventions as such, they might face challenges of adherence and compatibility to relevant medical evidence.

Therefore, this baseline study was aimed at examining patients' and providers' perspectives on how mobile phones (especially using an app) can improve the prevention, management, and treatment of stroke in rural settings. The findings of this study are to facilitate the design of a mobile phone app as an intervention.

## Methods

### Study design

A cross-sectional study using a mixture of methods was applied. The approach enhanced the study with more than one source of data and triangulation from different data sources and methods.

### Study setting, population, and sampling

The study was conducted among two general hospitals, Kiwoko and Luwero which were purposively selected for logistical reasons. The former is a Uganda Protestant Medical Bureau hospital (not-for-profit) owned by the Luwero Diocese the chairing diocese of Ndejje University Foundation Consortium, while the latter is a public health facility. Kiwoko Hospital is located in a rural setting while Luwero Hospital is located on the Kampala-Gulu highway (peri-urban) making it easily accessible to many patients from the neighboring districts.

Quantitative data collected was for 7 years (2015–2021) because the computerized comprehensive health information management system in the selected districts became operationalized in July 2014. So, we could access data from the following year to 2021 when the study was conducted. The qualitative data sample comprised ten homogeneous stakeholders including health workers (working with stroke patients, hospital administrators, and district health officials). Purposive sampling was employed to select health workers, and it enabled the selection of a knowledgeable category of respondents. Nine potential stroke patients/caregivers/survivors were chosen from the community. We employed snowball sampling to easily identify potential stroke patients/caregivers/survivors. The first participant was identified through the health worker who had attended the consultative meeting. Given that stroke patients had a hospital-based clinic, the first participant knew other potential participants and led us to them. Data from this category of participants was collected from Luwero Hospital since its patients were easily accessible. The eligibility criteria included (a) Health workers who directly attended to stroke patients for at least 6 months, (b) potential stroke patients/caregivers/survivors within the surrounding hospital communities irrespective of where they are getting health services, and (c) participants who could provide informed consent, (d) decision maker.

### Data collection methods and tools

Three data collection methods were employed. First, using the data extraction sheet that was developed to capture the total number of cases per year in Microsoft Excel, we extracted data for 7 years from the District health information management system (HMIS) for the two districts. This was done to indicate the trend of the disease. Working with the districts' HIMS managers, the lead author collected the data. Second, we held a Stakeholders' engagement and consulted with health workers. The consultation meeting involved getting their views/perceptions and input relating to using ICT in managing, preventing, and treating stroke. This was a 1-day meeting with follow-ups done through phone calls and emails. A consultative guide that was developed after analyzing quantitative data was used during the meeting. Audio recording, note-taking, and memoing were used during the meeting. The meeting was conducted in English by the three authors. Third, we conducted interviews with potential stroke patients/caregivers/stroke survivors in their homes. These were conducted by a research assistant trained and experienced in qualitative research. The participants were identified from Luwero Hospital. A semi-structured interview guide was used, and the discussions were audio-recorded. Seven out of the nine interviews were conducted in the local dialect (Luganda). The interviews were on average 1 h long and were audio-recorded. We achieved data saturation with both participant categories. Theme saturation was achieved among the second category of participants by first interviewing five participants, conducting an initial analysis, and then returning to the field/community and collecting more data from four participants.

### Data analysis

Quantitative data was entered in Excel sheets and descriptive analysis was run using Microsoft Excel version 10. Analysis results are presented in a figure. The descriptive inductive method was used to analyze qualitative data. Qualitative from key stakeholders was transcribed 1 day after the meeting by the lead author. The research assistant transcribed daily the data from semi-structured interviews in English and the two co-authors verified the English transcriptions. The two authors are fluent in both the local dialect and English. Audio recordings were played to give more clarity and meaning and ensure the accuracy of the notes and memoes. Data coding was done manually after transcription. Two researchers (*names withheld for blinded review purposes*) independently memo in the margins of the written transcripts to identify common phrases and words after the similarly coded text was merged and information re-written in a summarized manner. The similarities and differences between codes were analyzed to maintain the meaning of the data. The researchers then reviewed the final set of sub-codes to ensure that they answered the research questions. Since the different data sources were analyzed independently, the investigators integrated the data from the different sources to merge and compare results after the initial analysis. This resulted in data interpretation and discussion including identifying convergent and divergent issues from the data.

### Ethical considerations

Institutional Review Board approval was sought from Uganda Christian University (Mukono) No. UCUREC-2021-249. At each level of data collection, informed consent was sought from participants.

## Findings

### Participants' demography

Nine participants (63% males) attended the stakeholder engagement meeting. These included one district health officer, two hospital administrators, and six health workers. We also interviewed four patients, three caregivers, and two-stroke survivors from the targeted districts and 56% were females. All participants had cellphones, knew how to read, and write Luganda, and were residing in rural and semi-urban settings.

### Stroke cases in targeted health facilities

**Figure 1:**
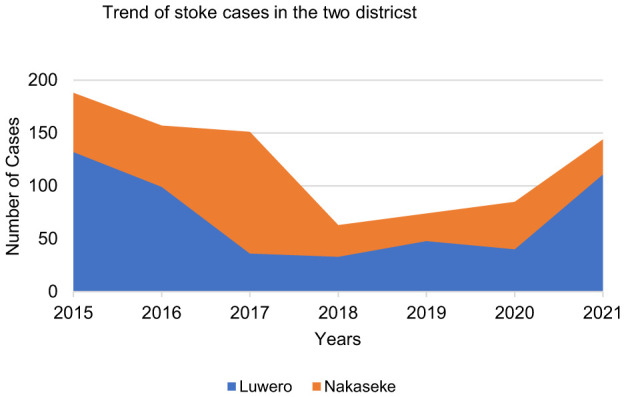
Summary of the stroke cases in the two selected districts.

The data collected was from the 2015 to 2021 calendar years from the Luwero and Nakaseke districts. A summary per district is presented in Figure 1.

The results above showed that none of the districts recorded 200 stroke cases annually throughout the study period. The results were presented to stakeholders and they discussed why there were seemingly few cases within the districts, yet stroke accounted for the top ten hospitalization cases.

“*You know stroke is an outcome of other diseases like hypertension, diabetes, HIV/AIDS, sickle cells, and others. So, what we have here are actual numbers of stroke patients, but many people with the underlying risk factors are potential stroke patients if not well managed” (Male SH 3)*.

### Current education platforms for stroke and the related underlying risk factors

The stakeholders decried the general lack of information about stroke disease among the majority of the population they serve. Generally, both the educated and illiterate had no information about stroke. The demand and supply side results indicated that the stroke survivors, their caretakers, and maybe people who have ever had a stroke patient are the only “common” people with information about stroke.

“*People don't have much information except those who visit the clinic and are diagnosed then they can know about that condition. Even the educated ones do not know much about this. Those with patients or the infected are the only ones who can be able to get this knowledge. ……... So those who know have been victims or caregivers” (Male SH 2)*.

“*…… during my master's thesis, I had the same problem where there were about 8 in 10 adult patients who didn't know that they were having hypertension until they were diagnosed” (Female SH 7)*.

“*We normally get this information from the hospitals given by the health workers, from families who had stroke patients or those who survived - that is if we get to know them. Not everyone has this information” (Female Caregiver 2)*.

Information about stroke, risk factors, and underlying causes was mainly given during the education sessions of those suffering from stroke underlying diseases and not every patient who visits the facility. The education messages are not structured to address stroke directly but rather those underlying diseases and their risk factors. Patients and caregivers collaborated on this finding. Sadly, some caregivers noted that although those sessions were conducted in the local dialect, sometimes they did not understand the “language” used by health workers. Implying that health workers were using technical terms that are neither in the local languages nor easy to understand.

“*And because nowadays stroke is common patients are educated about the risk factors and how to mitigate them. So, they educate them about diabetes, hypertension how it comes, and how to manage it. And with sickle cells, we normally educate the affected patients and the caregivers because many of them feel that they are bewitched” (Female SH 6)*.

“*Information is always received late since in most cases it's passed to them when people are already sick. Moreover, some information does not apply to particular patients since it's too general” (Male Recovering patient 1). People should be informed about strokes and take precautions before they get sick” (Female Caregiver 1)*.

“*Patients/caregivers sometimes do not understand doctor's language, they don't even ask questions, and they don't know what to ask” (Male Caregiver 1)*.

### Perception of the use of an app to get education/information about stroke

All patients, caregivers, and survivors thought that using an app to treat, manage, and prevent stroke was a wonderful idea. Among others, the intervention was seen as one that would help them save time and money in getting screened, getting educated, and going for refills.

“*It is a good idea because you get the info without spending a lot of time and finances. You don't need to move to the hospital all the time” (Male Recovering patient 4)*.

“*It will help since it saves time and is not costly, especially with reminders and closing the knowledge gap” (Female Caregiver 1)*.

### The target for stroke information

Although older people experience stroke more than their young counterparts, the message about stroke should target everyone. This was because the underlying risk factors like being diabetic or hypertensive including not adhering to related prescribed medication, accidents, and their associated trauma, having sickle cell, and others exist among all ages. This view was held by all participants.

“*……so, the target should be everyone because stroke can happen anytime to anyone. Though there are those with underlying diseases like hypertension who are more at risk, stroke can occur at any time for various reasons. So, everybody needs to know this information, and therefore, all should be targeted and encouraged to go for routine checkups” (Female SH 7)*.

“*All people should be sensitized. Address the public about the existence of the disease and avail facts about it irrespective of current people's health condition” (Male Caregiver 1)*.

### Relevant content/information for the educational stroke app

All participants recommended three information sections. First, the early warning signs and indicators of the stroke disease, capturing information for those with and with no underlying conditions. Second, the underlying factors/causes; could be generally spelled out but include genetic risk factors and the traditional ones that are modifiable like underlying diseases, alcohol consumption, and smoking along with nonmodifiable factors. Third, the prevention information; capturing simple measures to prevent or reduce the risks. All this information should be very precise and simple to understand by people of all education levels.

It was emphasized that self-management was vital in chronic care. Noting that some patients buy machines to monitor their health and need to be supported through reminders, healthcare providers indicated that some patients do not return for further examination after their first visit especially when they present cardinal signs at such a time. Thus, suggested that the app could have a section for reminding patients about their next appointment, drug refills, time for taking medication, blood pressure checkups for the patients, and where to get health services by linking Google Maps to all the networked facilities. Additionally, since patients do not know where to get stroke-related services, the app could influence stroke clinic days in health centers IV and hospitals, stating that lower facilities screen for risk factors and refer patients.

“*At our facilities, there are few doctors and many patients. This creates psychological pressure and disorder for both patients and health workers. That is why if appointments are made, you know when to go and are assured of being attended to at that particular time” (Female Caregiver 2)*.

“*So, the substantial number of people struggling with stroke are the people who have defaulted on medication. This app once developed will remind the patients of their next visit” (Male SH 8). Yes, there should be a reminder for the patients and the caregivers on appointments (Male SH 3)*.

“*There are possibilities of forgetfulness and inconsistency so, reminders can be helpful” (Male Caregiver 1). “There must be a continuous program alerting them and giving them information regarding their health about stroke and its effects. Some ways of reminding the caregivers about the patient's treatment and care” (Male Survivor 2)*.

“*They also get to know where to get the service…” (Male SH 8). “…. information is important for one to know who gives out that service and when it's offered so that the patients don't just guess where to go and when…” (Male SH 5). “The biggest challenge is that many patients don't know where to seek related services” (Female SH 1)*.

The app could be designed to have a section where the patient can make an appointment online, and the information gets to that specific health facility. Although it was noted that generally, patients in the two hospitals could just walk in at any time to receive the required services, it was observed this could be changed for people using the app/intervention. Stating that people generally have poor health-seeking behaviors, making appointments might improve their attendance to checkups. They would come to the facility knowing they would not waste their time waiting to be attended to.

Other issues regarding the information were: (i) the need for standardized measures, advising that literature should be reviewed to understand and use the Ugandan standard measurements, and there was no need to reinvent the wheel about measurements, (ii) developing a user-friendly app since many people do not like reading, and others cannot even read. For example, for those trying to predict their health status and checking themselves if they are at risk of stroke, simplified answer scorecards with yes/no should be used. Likewise, prioritize what information to give so as not to overload users with information at once.

### Potential challenges related to the planned intervention

#### Availability and accessibility of healthcare services

Most government facilities already have health system challenges of drugs and medicine stock-out, providers' absence at facilities, healthcare's negative attitudes, and non-functioning equipment among others. All these factors might demotivate and hinder those who potentially are at risk and need to confirm their health status. So, this has to be noted if such facilities are targeted for the intervention.

Relatedly, as people will be educated and easily determine their potential risks, the resultant effect will be for them to come for screening which might contribute to an increase in the number of people being served. This might also increase the number of patients for treatment. Although this might be a good thing, an increase in the utilization of services will exacerbate the inadequacies in the healthcare system due to the highlighted challenges.

The health workers advised that the introduction of this intervention should be done progressively. The old system should continue to run alongside the new one, and this will enhance the organic growth of the intervention in the process, and it might grow and “shallow” some of the outstanding challenges currently faced.

#### Opinions about demand side challenges

All participants had a mobile phone and estimated that eight in ten families within their settings also had a cell phone. It was noted that such an intervention requires the use of the Internet which is very expensive for many potential patients. Moreover, although the majority of families might have a mobile phone, a handful have smartphones as such the design of the intervention should consider this fact. These challenges were echoed by all participants.

“*The App can be helpful though we have challenges with the smartphone usability. First, not all people have smartphones. Smartphone operation is difficult because of the language” (Male Recovering patient 3)*. “*The app might be helpful, but some people ignore the smartphones and do not know how to use them” (Male Caregiver 1). “The techno-knowledge of phones is complicated – Language” (Male Survivor 2)*.

All participants reiterated the need to train/educate potential users about the whole intervention, and specifically how to use the app before its introduction.

It was also noted that although current patients had clinic days and visited the health facility on specific days, there was a challenge of congestion, and sometimes they were not served on time. Emphasizing that making appointments might solve this challenge, although the participants asked health workers to solve the challenge of spending a lot of time at health facilities.

“*you can only come on the specified days and time and there is no special attention, congestion. There are no timely services due to the congestion with other patients” (Female Caregiver 1)*.

#### Other challenges

The people need to trust the intervention before they can plan to use it. People might fear a breach of their personal information especially if such data is required at the time of registration/downloading the app. Moreover, those diagnosed with risk factors might fear using the app if they are not sure of the safety of their personal information. To improve trust in the intervention, participants suggested working with existing structures of healthcare providers in targeted facilities, community health workers, and religious institutions to inform people about the intervention.

## Discussion and conclusion

We examined how mobile phones can improve the prevention, management, and treatment of stroke in rural Uganda. To the best of our knowledge, this is the first study in Uganda to assess patients' and providers' perspectives on adopting ICT in stroke-targeting rural settings. The decision-makers involved in this study are well-suited to embed the results and ensure maximal support of the intervention.

Just like previous studies that have found mobile phones to be useful in providing information to stroke patients, self-management, and future patients' education (Nam et al., 2013; Mosa et al., 2012), participants in this study suggested three major types of information to be included in this intervention: warning signs and indicators, underlying causes of stroke and prevention measures. Evidence shows poor public knowledge of stroke warning signs worldwide (Jauch et al., 2013). Participants suggested the inclusion of reminders for patients for different aspects of stroke management anticipating that this might contribute to improved health-seeking behaviors of both the patients and those at risk. This aligns with experiences elsewhere that support that smartphones/apps can provide real-time feedback to users, are effective platforms that support changes in health behaviors, and allow individualized content (Fanning et al., 2012; Klasnja et al., 2011).

People in rural settings possessing mobile phones were found in other studies within the region (Hellström, 2010). Likewise, the fact that all participants from the health user side supported the use of mobile phone intervention indicates a substitute for physical access to health facilities for some aspects of stroke management and prevention and may provide an affordable alternative to accessing some of the relevant services to the rural people. However, the study findings revealed several contextual, health system, and economic aspects relevant to planning the intervention. These were related to the healthcare services availability and accessibility, limited knowledge of using phones and the fact that few people might have smartphones, the use of the internet which is expensive, and distrust of the intervention especially if participants are not confident of the privacy of their information provided while using an app. These challenges have been documented in previous studies (Haller et al., 2009; Maokola et al., 2011; Burdette et al., 2008; Oehler et al., 2010; Johansson et al., 2010; van Ettinger et al., 2010). All these are important issues that have to be considered/addressed while designing the intervention. Previous scholars (Haller et al., 2009; Maokola et al., 2011) have noted several other challenges related to the use of apps and smartphones in healthcare. These include low phone battery life, potentially inefficient patient-physician interaction, small screen size, and breach of data privacy and security among others. Although they did not come out in the results, these challenges are relevant in the context where data was collected. The participant may have limited experience with phone healthcare which is why such relevant challenges were not highlighted during the discussion.

Although the study's intervention is to target beneficiaries, available evidence shows numerous existing apps applied by healthcare professionals (Mosa et al., 2012). Health professionals were using these apps for disease diagnosis (Burdette et al., 2008; Oehler et al., 2010), drug reference (Johansson et al., 2010; Oehler et al., 2010), and general healthcare applications (van Ettinger et al., 2010). This enforces the need for more user-friendly apps on the demand side of the healthcare system.

There are limitations to this study. First, the qualitative part of the study focused on individuals' experiences and opinions from the demand and supply side of the healthcare system which might be biased. However, triangulation by category of participants through comparative analysis was conducted. Second, five out of the nine interviews for stroke patients, survivors, and caregivers were conducted in the home environment, and the rest were in health facilities. There were interferences during the interviews by various activities and people which could have affected the trustworthiness. Third, participants were from rural/semi-urban settings, so findings might not be relevant in an urban setting.

There is an increase in the use of mobile phones in the delivery of health services in developing countries due to their increased accessibility, especially in rural areas. Both health workers and patients can benefit a lot from the different functions that these phones can serve ranging from information dissemination to the management of health services and diseases. It is hoped that the use of mobile phones will fill some of the gaps in primary health care, especially for medical conditions such as stroke. These baseline findings support the possibility of mobile phone intervention as an important instrument to improve the prevention, management, and treatment of stroke in rural Uganda. The use of an app has Great potential to impact stroke prevention and management outcomes not only in the proposed regions but throughout the country. Many challenges might accompany the design and use of ICT in health care delivery including barriers to accessing digital infrastructure such as internet and low digital literacy in rural areas. However, these challenges have to be addressed as the intervention is designed. Therefore, careful planning and continued stakeholder engagement in the next phases are essential for the success of the implementation and sustainability of the intervention.

## Data Availability

The data that support the findings of this study are available on request from the corresponding author [PKN]. The interview transcripts are not publicly available because they contain information that could compromise the privacy of research participants.
