# Supplementary material for: Adopting information and communications technology in the control, prevention, and management of stroke: perspectives from patients and providers in Uganda
Source: Front Stroke. 2024 Dec 18;3:1440047. doi: 10.3389/fstro.2024.1440047 (PMC12802623; doi:10.3389/fstro.2024.1440047)
Supplement: Supplementary file 1 [file Supplementary_file_1.docx]

**Consultative guide with health workers**

**Introduction:**

Stroke is among Uganda’s 10 causes of death ranks 9^th^ and is a major cause of chronic illness. In Uganda, not much study has been done on stroke and its management. People lack information about this, and they don’t know where to get such information. And those who have this information are the victims and the caregivers of those victims. We also wanted people to know where they could get help if they wanted such help. We want to interact with you about the feasibility of using ICT in stroke prevention, management, and care. This is a baseline data collection, which will inform the design of an intervention.

1. From the quantitative data we collected, there are less than 200 cases per year in each of your hospitals, and yet stroke is among the 10 leading causes of death in Uganda. Why this discrepancy?
2. How do people get information about risk factors and how to prevent stroke? Probe: for education approaches within health facilities, the kind of information given,
3. One way to solve the information gap for people at risk is to develop an app. What kind of information should this app give? Probe: details about exact information such patients may need.
4. What challenges are likely to be faced with this intervention?

Semi-structured interview guide for patients/caregivers/stroke survivors

1. Where do people get education about stroke from (Probe: challenges with these channels)
2. What do you think about having an App as part of stroke prevention, management, and care?
3. What could the app be used for – what kind of services/information could the app provide?
4. What challenges are likely to be faced in using an App system in the prevention, control, and management of stroke? (Probe: language, access to phones, cultural constraints, others)
5. What is the best approach to introducing the idea of an app with all the different content highlighted above (Probe: at community level & health facility level)
